# Supplementary material for: WheatCENet: A Database for Comparative Co-expression Networks Analysis of Allohexaploid Wheat and Its Progenitors
Source: Genomics Proteomics Bioinformatics. 2022 Jun 1;21(2):324–36. doi: 10.1016/j.gpb.2022.04.007 (PMC10626052; doi:10.1016/j.gpb.2022.04.007)
Supplement: Supplementary File S1 [file mmc1.docx]

**Case study**

**Using WheatCENet to predict unknown gene functions in diploid and polyploid wheat**

Cytochrome P450s are the largest family of proteins in higher plants [1] and are known to respond to environmental and developmental cues and tissue and subcellular localization [2]. In the comprehensive comparative analysis of CYP450 in hexaploid wheat and maize, both species have potential functional separation and tissue-specific expression [3]. By comparing the protein sequences of hexaploid AABBDD and its progenitors with wheat protein sequences of CYP450 (http://drnelson.uthsc.edu/cytochromeP450.html), we further identified their P450 domains using PfamScan (https://www.ebi.ac.uk/Tools/pfa/pfamscan/). After filtering, 1564, 823, 462, and 489 full-length *CYP450* genes were found in AABBDD, AABB, AA, and DD, respectively (Table S5). According to the standard naming rules, there are 43, 42, 41, and 43 CYP450 families in AABBDD, AABB, AA, and DD, respectively. In A-type, *CYP71* belongs to the family with the largest number of genes among the four species. In non-A-type, the *CYP96* family accounts for the largest numbers of genes in AABBDD and AA, while the *CYP72* and *CYP51* families account for the largest numbers of genes in AABB and DD, respectively (Figure S7A).

Furthermore, the subgenomes of AABBDD and AABB were classified into A-type and non-A-type families, and the number of A-type gene families was higher than that of non-A-type gene families in all species and corresponding subgenome genes. The gene numbers of A-type and non-A-type families basically conform to the proportion of 1:1:1 or 1:1 in the subgenomes of AABBDD and AABB (Figure S8B). Because both diploids and polyploids contain roots, leaves, and grains, we mainly looked for the expression profile clustering of these three tissue-specific genes. Ultimately, we found 34, five, and three CYP450s in the roots, leaves, and grains, respectively, which were specifically expressed **(**Figures S7 and S8**)**. Of these, *CYP71X5* was expressed specifically in grain in all species (Figures S8 and S9). *CYP71X5* had five, three, one, and one member in AABBDD, AABB, AA, and DD, respectively (Table S5). There is no corresponding research on *Arabidopsis*. We speculate that *CYP71X5* may be related to grain development.

By searching using the TraesCS6A02G180100 gene (one of the genes of *CYP71X5* in AABBDD) in our database, four of the five genes of *CYP71X5* in AABBDD were included in CFinderM000360 (Figure S9A), which consists of 51 nodes, and the most significant annotation of the module was ‘embryo sac egg cell differentiation’ (GO:0009560) (Figure S9B). Some genes are related to grain development in CFinderM000360: *mother of FT and TFL1* (*TaMFT*) has been confirmed to regulate seed germination in wheat [4]; *Prolamin binding factor* (*PBF*) may influence embryo size and endosperm starch synthesis [5], ADP-glucose pyrophosphorylase (*Agp2*) controls the rate-limiting step in the starch biosynthetic pathway, have starch accumulation and expression peak at 21 and 15 days post-anthesis (DPA) in wheat grains [6,7], homogentisic acid geranylgeranyl transferase (*HGGT*), which catalyzes the committed step of tocotrienol biosynthesis as the primary form of vitamin E in seeds of most monocot plants [8], and *TaKO-B* is related to gibberellin synthesis [9] (Figure S9A). We performed a cis-element analysis of the 3 kb upstream region of all genes in the module and found that some seed-specific motifs, such as GCN4_motif and Skn_1_like_motif, are significant (Figure S9C**)**. The expression profile of the module shows that the positive genes co-expressed with *CYP71X5* are specifically expressed in grains (Figure S9D**)**. Taken together, *CYP71X5* may be related to grain development, and genes in the CFinderM000360 module may have specific functions in grain development.

**References**

[1] O'Keefe DP, Tepperman JM, Dean C, Leto KJ, Erbes DL, Odell JT. Plant expression of a bacterial cytochrome P450 that catalyzes activation of a sulfonylurea pro-herbicide. Plant Physiol 1994;105:473−82.

[2] Bolwell GP, Bozak K, Zimmerlin A. Plant cytochrome P450. Phytochemistry 1994;37:1491−506.

[3] Li Y, Wei K. Comparative functional genomics analysis of cytochrome P450 gene superfamily in wheat and maize. BMC Plant Biol 2020;20:93.

[4] Nakamura S, Abe F, Kawahigashi H, Nakazono K, Tagiri A, Matsumoto T, et al. A wheat homolog of MOTHER OF FT AND TFL1 acts in the regulation of germination. Plant Cell 2011;23:3215−29.

[5] Orman-Ligeza B, Borrill P, Chia T, Chirico M, Dolezel J, Drea S, et al. *LYS3* encodes a prolamin-box-binding transcription factor that controls embryo growth in barley and wheat. J Cereal Sci 2020;93:102965.

[6] Zhang XW, Li SY, Zhang LL, Yang Q, Jiang QT, Ma J, et al. Structure and expression analysis of genes encoding ADP-glucose pyrophosphorylase large subunit in wheat and its relatives. Genome 2016;59:501−7.

[7] Ainsworth C, Hosein F, Tarvis M, Weir F, Burrell M, Devos KM, et al. Adenosine diphosphate glucose pyrophosphorylase genes in wheat: differential expression and gene mapping. Planta 1995;197:1−10.

[8] Cahoon EB, Hall SE, Ripp KG, Ganzke TS, Hitz WD, Coughlan SJ. Metabolic redesign of vitamin E biosynthesis in plants for tocotrienol production and increased antioxidant content. Nat Biotechnol 2003;21:1082−7.

[9] Huang Y, Yang W, Pei Z, Guo X, Liu D, Sun J, et al. The genes for gibberellin biosynthesis in wheat. Funct Integr Genomics 2012;12:199−206.
