# Supplementary figures and images for: WheatCENet: A Database for Comparative Co-expression Networks Analysis of Allohexaploid Wheat and Its Progenitors
Source: Genomics Proteomics Bioinformatics. 2022 Jun 1;21(2):324–36. doi: 10.1016/j.gpb.2022.04.007 (PMC10626052; doi:10.1016/j.gpb.2022.04.007)

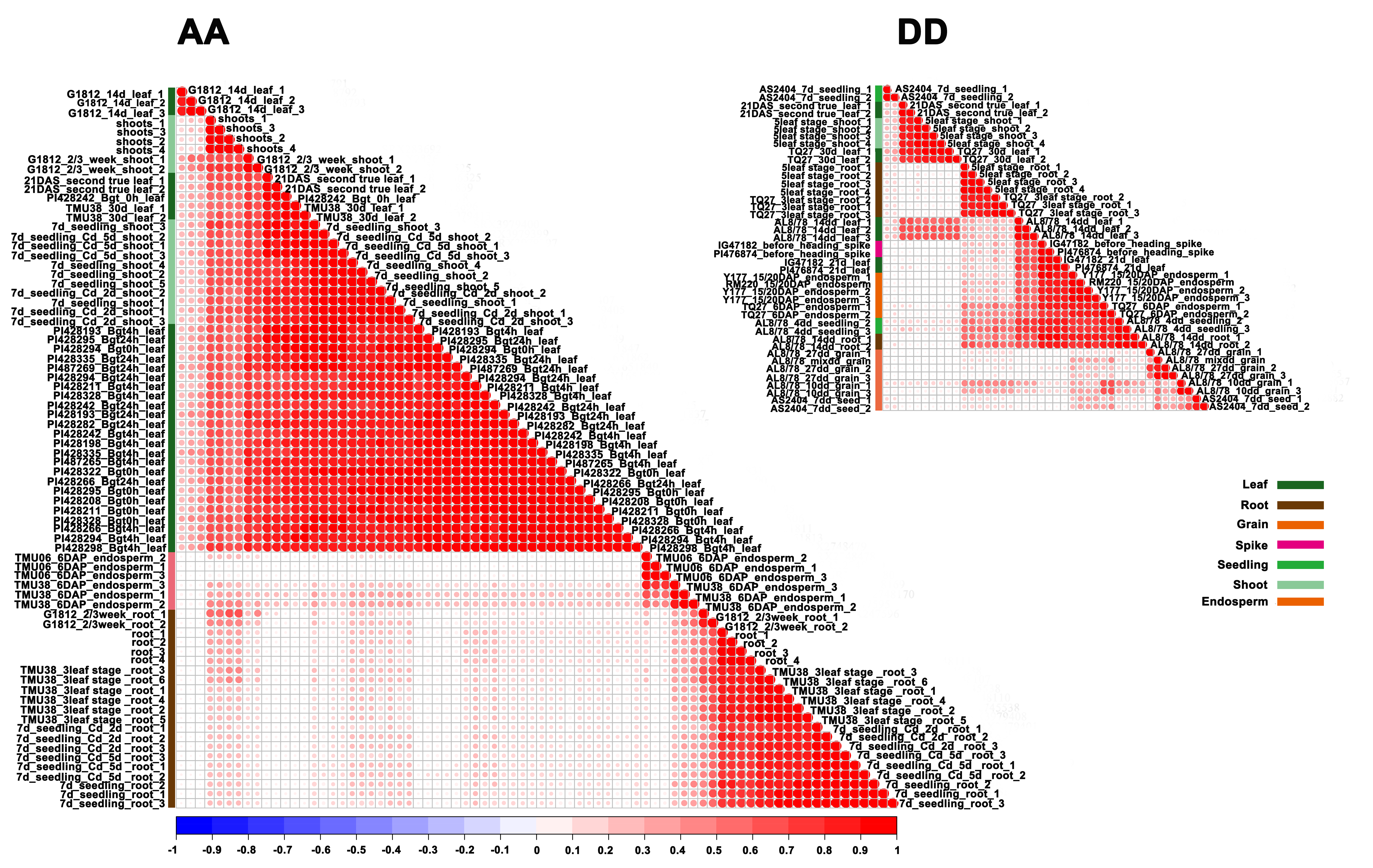

Supplement: Supplementary Figure 2 [file mmc8.zip › Figure S2.png]

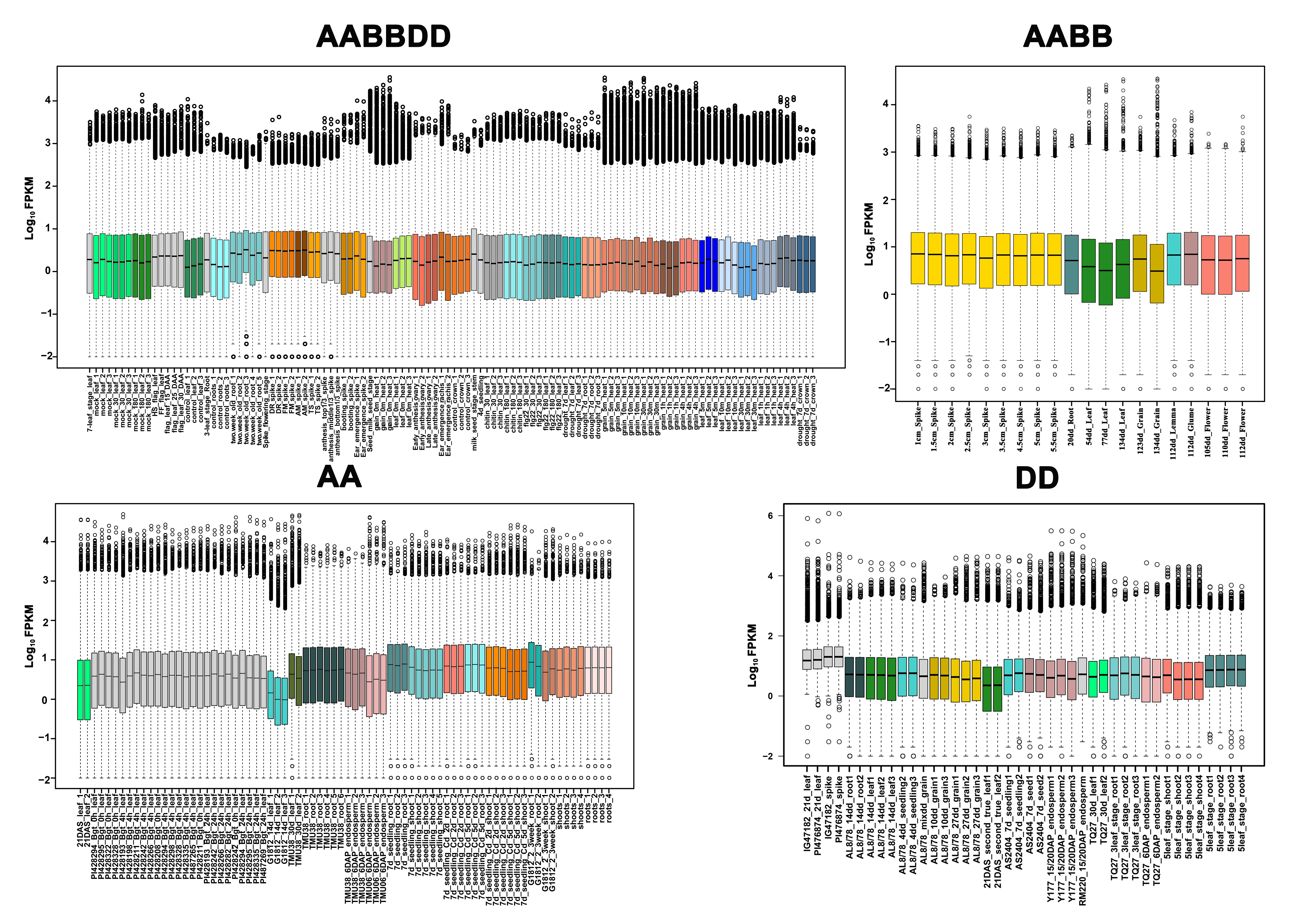

Supplement: Supplementary Figure 3 [file mmc9.zip › Figure S3_2022-5-24.png]

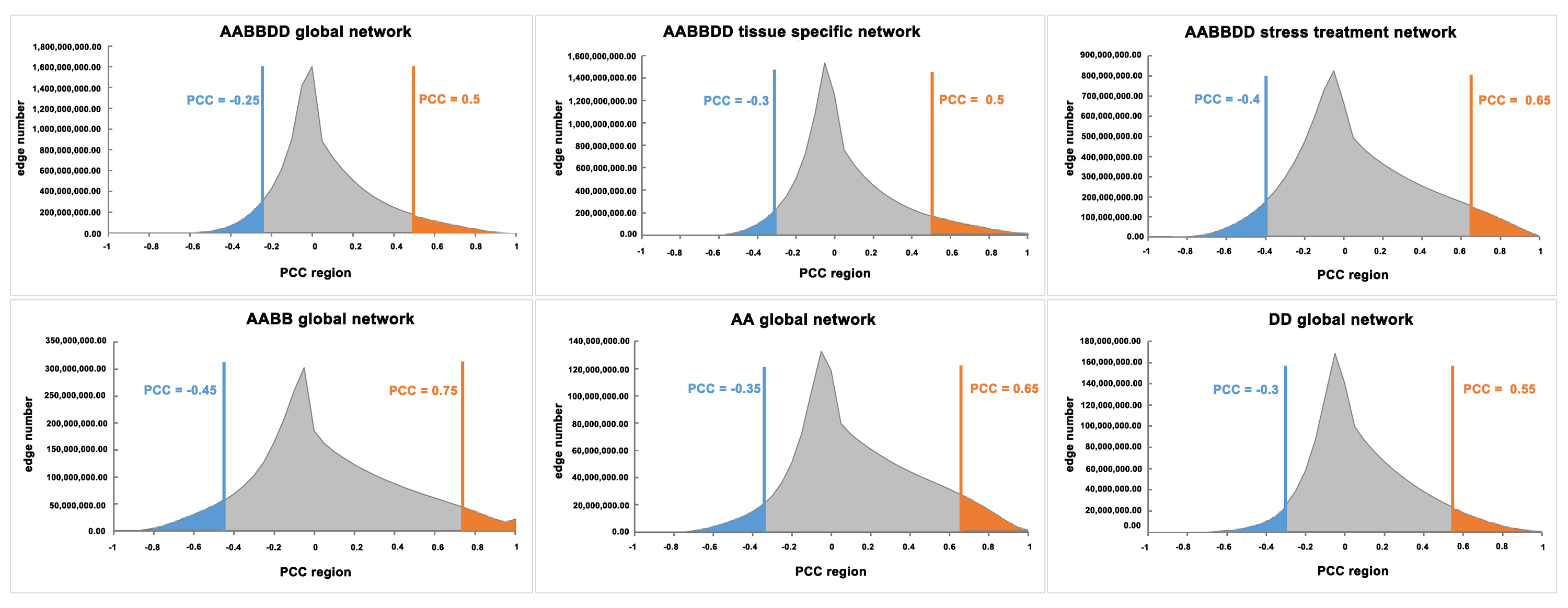

Supplement: Supplementary Figure 4 [file mmc10.zip › figure S4.png]

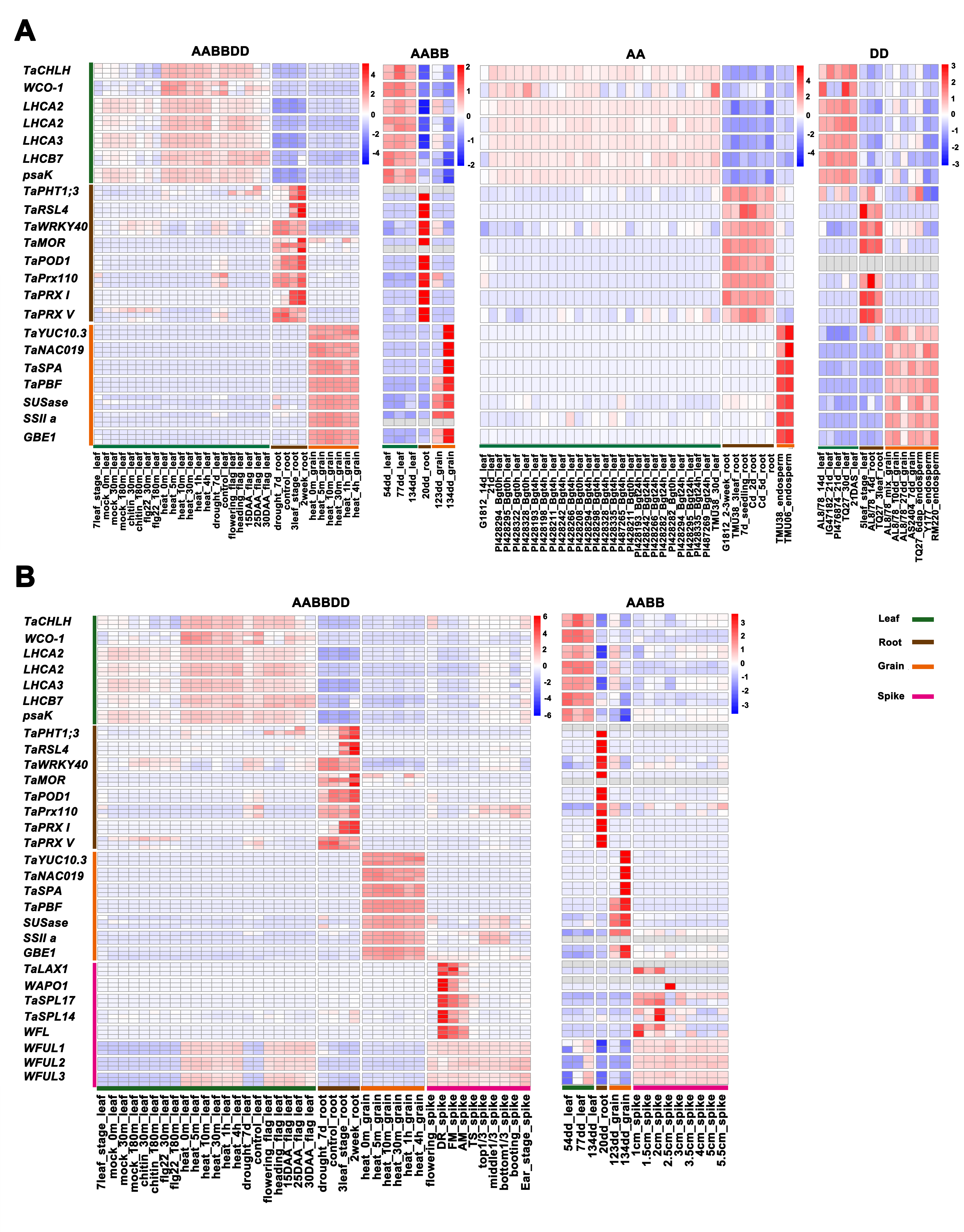

Supplement: Supplementary Figure 5 [file mmc11.zip › Figure S5.png]

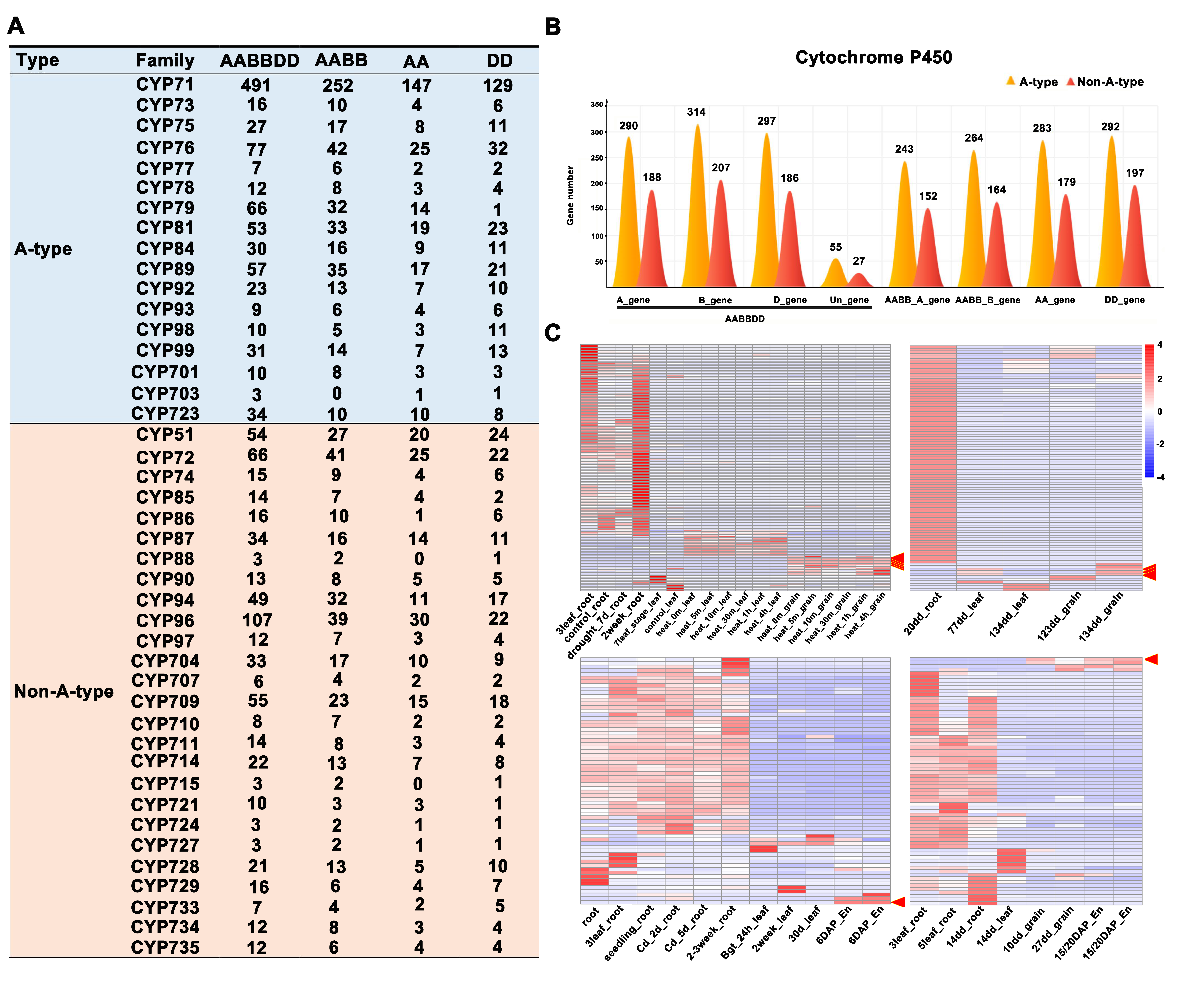

Supplement: Supplementary Figure 7 [file mmc13.zip › Figure S7.png]

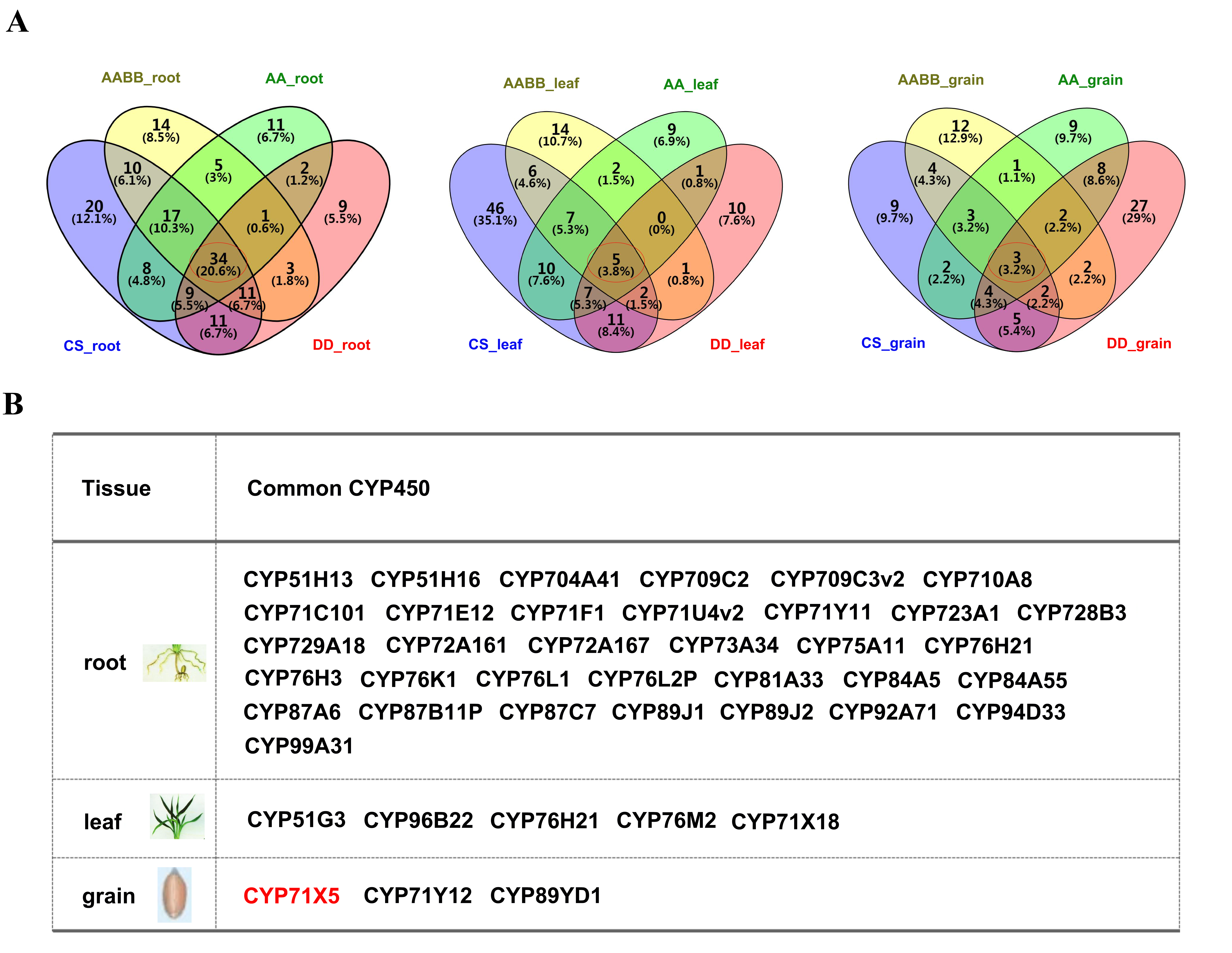

Supplement: Supplementary Figure 8 [file mmc14.zip › Figure S8.png]
